# A Partnership Among Local Public Health Agencies, Elementary Schools, and a University to Address Childhood Obesity: A Scalable Model of the Assess, Identify, Make It Happen Process

**DOI:** 10.3389/frhs.2022.816536

**Published:** 2022-07-12

**Authors:** Benjamin C. Ingman, Carla Loecke, Elaine S. Belansky

**Affiliations:** Center for Rural School Health and Education, Morgridge College of Education, University of Denver, Denver, CO, United States

**Keywords:** implementation science, research intermediaries, elementary schools, school health, rural, public health agencies

## Abstract

**Background:**

One pathway to addressing childhood obesity is through implementing evidence-based practices (EBPs) shown to promote nutrition and physical activity in K-12 school settings. Assess, Identify, Make it happen (AIM) is a strategic planning process to engage stakeholders in implementing EBPs in their K-12 schools. Local Public Health Agencies (LPHAs) are a potential partner to facilitate this process to a broader audience of rural school communities.

**Methods:**

A process and outcome evaluation design was applied in this study to examine the extent to which LPHAs effectively implemented AIM with rural/frontier schools in comparison to university staff. Data collection included post-meeting surveys completed by facilitators, a post-intervention interview with facilitators, a survey of school task force members at the end of the AIM process, and systematic documentation of the intervention.

**Results:**

***Reach***—Among the 26 eligible elementary schools, 18 (69%) agreed to participate.

***Effect***—In total, schools facilitated by LPHAs fully implemented an average of 4.0 changes per school, while schools facilitated by the university staff fully implemented an average of 3.7 changes.

***Adoption***—Among the five LPHAs in the target region, all five agreed to partner on the initiative, but some agencies were unable to identify sufficient personnel to facilitate all schools in their catchment area.

***Implementation***—(1) In total, 89 of 94 (95%) meetings scheduled by LPHA facilitators occurred. 47 of 48 (98%) meetings scheduled by the university staff occurred. (2) The university staff self-reported 93% of agenda items in the AIM process as “completely” followed while LPHA facilitators reported 41% of agenda items as “completely” followed. (3) Task force satisfaction with the AIM process and facilitator showed limited variance across LPHAs and university-facilitated schools.

***Maintenance***—Of the 16 school districts that agreed to participate in the school-based version of AIM, 9 (56%) also participated in a district-wide version of AIM 2 years later.

**Conclusion:**

AIM is an effective process for implementing EBPs in elementary schools when facilitated by LPHAs. Effective partnerships, a nuanced approach to fidelity, scalability considerations, and the role of technical assistance and training all contributed to the successful implementation of this LPHA-Elementary school partnership.

## Background

### Childhood Obesity, Schools, and the Role of Research Intermediaries

Childhood obesity rates have continued to climb over the last several decades across the United States, with higher rates of obesity among rural youth (1), Latinx youth (2), and youth living in poverty (3). Schools are situated to address these systemic inequities by promoting nutrition and physical activity (4). This is especially the case in rural communities, where schools are often considered the hubs of social and cultural activities (5).

The evidence for school-based practices and policies that promote students' physical activity (6), nutrition (7), and mental and behavioral health (8) continues to grow. Despite ongoing concerns about the efficacy of childhood obesity prevention programs (9), there are many practices reflected in the literature that have been shown to increase student opportunities for physical activity and nutrition in schools (10, 11). Evidence-based practices (EBPs) in K-12 schools that promote nutrition include cafeteria-based practices [e.g., offering healthy beverages and foods (12), placing fruits and vegetables earlier in the line (13), scheduling recess before lunch (14), using an “offer” rather than “serve” system (15)]; as well as practices outside the cafeteria [e.g., healthy food for class parties and rewards (16), regular access to water (17), and school store policies that promote healthy food and drinks (18)]. Increased physical activity in schools is linked to practices for physical education [e.g., using an evidence-based curriculum and equipment (19, 20)], environment [e.g., adequate indoor and outdoor facilities (21)], recess [e.g., not withholding recess as punishment, providing equipment and organized activities during recess (22)] classrooms [e.g., classroom activity breaks (23), standing desks (24)], and extracurricular activities [e.g., providing intramural or interscholastic sports (25)]. However, many schools have not implemented those practices or recommendations (26). This disconnect between research and practice, routinely documented in the fields of public health and healthcare (27, 28) are also reflected in the implementation status of practices and policies in K-12 schools (29, 30).

Research intermediaries, or organizations that help community-based entities learn about and implement EBPs (among other functions) (31), have made progress in facilitating the connection between research and practice in K-12 schools. In particular, leveraging practices of community engagement to facilitate the translation of EBPs to school environments has shown promise (32, 33). However, additional strategies are necessary to reach schools in rural, high-poverty settings where resources and research tend to be scarce (34). One pathway to address these gaps in knowledge and translation is through engaging school stakeholders in a process to implement EBPs in their schools. Such a process can reach more schools if additional organizations and agencies are identified and mobilized as research intermediaries.

### AIM (Assess, Identify, Make It Happen)

Assess, Identify, Make it Happen (AIM) is a strategic planning process to promote healthy nutrition and physical activity in K-12 schools. In this process, a task force of community stakeholders convenes to Assess the current status of evidence-based practices (EBPs) shown to promote healthy nutrition and physical activity, Identify EBPs to put in place, and Make it happen by implementing those EBPs. The 12-month process is facilitated by a trained and certified facilitator.

AIM was tested in rural, elementary schools using a pair-randomized control trial and demonstrated to be an effective strategy for promoting the implementation of effective school-based environment, policy, and practice features previously shown to increase students' physical activity and healthy nutrition (29). AIM schools made an average of 4.4 evidence-based changes per school with 90% still in place a year later compared to schools that used the CDC's School Health Index which made an average of 0.6 effective changes with 66% in place a year later. This first study demonstrated that AIM is an effective method of promoting the implementation of EBPs when facilitated by university staff working directly with rural communities. While these results bode well for the process itself, relying on university staff to implement AIM poses a challenge to scalability (i.e., relies on university-based personnel and considerable travel expenses). A delivery model in which individuals from rural communities facilitate the process in their own communities would greatly improve the scalability of AIM.

### Local Public Health Agencies

Local Public Health Agencies (LPHAs) were identified as entities well positioned to promote the scalability of AIM. Among the ten essential services of LPHAs are to: Communicate effectively to inform and educate people about health, factors that influence it, and how to improve it; Strengthen, support, and mobilize communities and partnerships to improve health; Create, champion, and implement policies, plans, and laws that impact health (35). These functions closely align with the purposes of the AIM process. Additionally, LPHAs are physically proximate to target populations, have considerable knowledge of the community, and prioritize addressing childhood obesity. Although LPHAs in rural/frontier regions may face challenges such as a lack of qualified staff, and limited access to training, information, and resources (36, 37), they are also well positioned to leverage local cultural assets and flexible structures for developing new productive partnerships and networks (38). Further, half of the 2,400 Local Health Departments/Agencies in the USA serve rural populations (39). This confluence of factors positions LPHAs as a promising pathway to scalability for school- and community-based initiatives. Others have been successful in partnering with LPHAs to implement school-based initiatives (40), although concrete assessments of implementation characteristics in applying such an approach are scant in the literature.

Partnering with LPHAs to facilitate the AIM process required important changes to several elements of the AIM process, facilitator training, and technical assistance (41). Specifically, this change in implementation model was coupled with the development of an AIM website, the revision of AIM meeting guides and materials, streamlining and automating labor-intensive aspects of the process (e.g., creating an automated survey and report generating system). For these reasons, an implementation science framework was adopted to evaluate not only the outcomes of the intervention, but also to describe key dimensions of implementation across the RE-AIM framework (42). This work contributes to discourse of implementation science that seeks to understand the effectiveness of interventions when implemented in real-world settings and provides additional perspectives on the factors that influence successful implementation (43).

The purpose of this study was to examine the extent to which LPHAs could effectively facilitate AIM with rural/frontier schools in comparison to university staff. The RE-AIM framework was used for this inquiry because it provides a systematic and comprehensive structure to evaluate interventions as implemented in complex, real-world settings.

## Methods

### Program Implementation

#### Program Description: AIM Process

The goal of the AIM process is to implement evidence-based practices (EBPs) for promoting student nutrition and physical activity in school settings. For each school participating in AIM, a task force of school stakeholders (including the school principal, classroom teachers, physical education teachers, school staff, food service directors, nurses, and parents) convenes for a series of meetings led by a facilitator trained and certified in the process. The AIM facilitator is provided a facilitator guide, which includes detailed agendas, activities, and talking points for each meeting, as well as tasks to complete between meetings. Before the AIM process begins, baseline data is collected *via* a three-module survey based on the School Environment and Policy Survey (SEPS) (29). This survey is completed by the principal, food service director, and physical education teacher and generates a Best Practice Report that provides the status (fully implemented, partially implemented, not implemented) of EBPs for nutrition and physical activity.

After the task force has been recruited and oriented to the process, they discuss strengths and challenges related to student health behaviors and school practices to promote student health. The task force also reviews the Best Practice Report to understand the current implementation status of nutrition and physical activity EBPs in the school and generate a list of potential changes to make to the school. This list of potential changes is later revised and clarified before final selections are made based on the importance of a change for student health and the feasibility of implementing it.

The task force engages in several planning activities to promote the successful implementation of the selected changes. This includes planning to get approval and buy-in for changes, identifying individuals to champion changes, creating a task-oriented timeline for implementing changes, and planning for sustainability. The task force convenes for a final meeting to review progress in implementation, and plan any next steps for the group, such as checking in on implementation or transitioning to a wellness team.

The AIM process was implemented with two separate cohorts and revised between cohort 1 (eight schools) and cohort 2 (10 schools) based on feedback from facilitators and task force members. The most significant revision was the amount of time dedicated to AIM meetings and activities; the number of meetings was reduced from 9 to 7 meetings, and the length of meetings was reduced from 120 to 60–75 min (see Table 1).

**Table 1:** AIM process meeting descriptions.

**AIM Process for Cohort 1 (2014–2015)** ***9 meetings, 120 min each 9 (6) schools***		**AIM Process for Cohort 2 (2015–2016)** ***7 meetings, 60–75 min each 10 (7) schools***	
**Meeting title**	**Meeting description**	**Meeting title**	**Meeting description**
**ASSESS**		**ASSESS**	
**1. Getting started**	Introduction to AIM, school snapshot Pt 1: strengths	**1. Looking for opportunities**	Identify strengths and opportunities related to healthy eating and physical activity in different parts of the school (e.g., cafeteria., classroom, before/after school)
**2. Looking for opportunities**	School snapshot Pt 2: opportunities, best practice report, list of possible changes	**2. Investigating best practices**	Review best practice report, make a list of possible changes
**IDENTIFY**		**IDENTIFY**	
**3. Evaluating change possibilities**	Rating importance and feasibility	**3. Identifying changes**	Rate importance and feasibility, select changes
**4. Selecting changes**	Review importance and feasibility, select changes		
**MAKE IT HAPPEN**		**MAKE IT HAPPEN**	
**5. Planning for approval and buy-in**	Create action plans: Focus tasks on getting approval to make changes and building buy-in among stakeholders	**4. Building support for changes**	Action Planning: Tasks to get approval and build buy-in for changes
**6. Planning for implementation**	Create action plans: Focus tasks on nuts and bolts of implementing practices	**5. Planning for implementation**	Action Planning: tasks to put changes in place and sustain them over the long term
**7. Planning for sustainability**	Create action plans: Focus on tasks to sustain changes over time; create timeline for implementing practices	**6. Wrapping up**	Create timeline for implementing changes and assign tasks, plan for summer
**8. Checking our progress**	Assign remaining tasks, plan for summer	**7. Checking in**	Check in to document progress and keep things on track
**9. Moving forward**	Check in the following fall to document progress and keep things on track		

#### Program Setting

This study took place from 2014 to 2016 in a rural/frontier plains region in Colorado encompassing seven counties and 15,962 square miles (larger than the state of Maryland) that includes the lowest county health rankings and highest childhood poverty rates in the state (44).

#### Program Recruitment

Project staff recruited LPHAs and schools through in-person visits at each site during the academic school year preceding the intervention. School recruitment meetings were typically attended by the school principal and physical education teacher. Schools received $4,000 to complete the AIM process. LPHA recruitment meetings were attended by agency directors and staff identified as potential AIM facilitators, who were in most cases nurses. Informational flyers explaining the AIM process and Memorandums of Understanding were key artifacts used during recruitment efforts. LPHAs were remunerated at a rate of 10% FTE of the facilitator per each school facilitated (e.g., one school facilitated through AIM by an LPHA employee earning $50,000 resulted in a $5,000 payment to the LPHA).

Local Public Health Agencies staff also participated in a readiness assessment interview during the recruitment phase, which provided an opportunity to discuss their motivations and reservations to participating. LPHAs noted the shared priority of addressing obesity (all five included obesity in their most recent Health Assessment Plans) and the potential benefits of closely collaborating with schools in their service area.

#### Training and Technical Assistance for LPHAs

Local Public Health Agencies directors designated staff to facilitate the AIM process. LPHA staff were trained through a 5-day training in August and a 1-day booster training midway through the school year. Two facilitators who worked with both cohort 1 and cohort 2 attended a 1-day training focused on revisions from the previous year in lieu of attending the 5-day training a second time. Ongoing support to discuss progress and answer questions consisted of monthly conference calls among facilitators and university staff, and individualized *ad hoc* technical assistance [see (45)].

### Process and Outcome Evaluation Design

This study used a process and outcome evaluation approach to monitor and evaluate the implementation of the AIM process (46). Process evaluation efforts, which were guided by the RE-AIM framework (42), began with the recruitment of LPHAs and schools and ended 6 months after all participating schools had completed the AIM process. Outcome evaluation was focused on the implementation of evidence-based practices in participating schools and general satisfaction with the AIM process and facilitators. The RE-AIM framework was selected to guide data collection because it attends to various factors of implementing real-world public health interventions (Reach, Effect, Adoption, Implementation, Maintenance; see Table 2). This study was approved by the Colorado Multiple Institution Review Board.

**Table 2:** RE-AIM constructs and evaluation metrics.

**RE-AIM Dimensions (42)**	**Evaluation metrics in this work**
***Reach***. Proportion of the target population that participated in the intervention	• Number and demographic characteristics of participating school districts in the target region
***Effect** (or Efficacy)*. Success rate if implemented as in guidelines/protocol	• Number of physical activity and nutrition evidence-based practices fully implemented, partially implemented, planned for implementation, and not implemented
***Adoption***. Proportion of settings that adopt the intervention	• Number and characteristics of LPHAs in the target region implementing AIM
***Implementation***. Extent to which the intervention was implemented as intended	• Number and length of meetings facilitated • Facilitator time spent preparing and feelings of preparedness • Facilitator fidelity to facilitator guide • Extent of idea sharing and tension noted during meetings • Taskforce satisfaction with AIM process and facilitators
***Maintenance***. Extent to which a program is sustained over time	• School district participation in a subsequent version of AIM • Anecdotal continuation of wellness teams

### Data Collection

#### Post-meeting Surveys (AIM Facilitators)

All AIM facilitators (LPHA staff and university staff) completed a post-meeting survey at the end of each AIM meeting. These surveys included attention to logistical aspects of the meeting (date, time, and length of the meeting); facilitator preparation; fidelity to the meeting guide; task force dynamics (member participation and tension during the meeting); and feedback about the meeting agenda and process. There was an average of 33 items per post-meeting survey. Implementation status of changes was included in the final AIM meeting survey. These surveys were completed with a 100% response rate.

#### Post-intervention Interviews (AIM Facilitators)

All AIM facilitators participated in a semi-structured interview at the end of the intervention. These interviews were held in person at the health agency office or in a community setting and focused on LPHA facilitator perspectives on four topics: (1) facilitation of the AIM process at the school, (2) partnership with the university team, (3) impacts on the agency or its personnel, and (4) suggested improvements to the AIM process.

#### Post-process Survey (AIM Task Force Members)

Those participating in the AIM process as members of school task forces completed a 53-item survey at the end of the AIM process. Key topics included in this survey were perceptions of the facilitator and overall satisfaction with the AIM process. In total, 80 task force surveys were completed, representing a 100% response rate for task force members in attendance at the final AIM meetings.

#### Process Documentation

Other data, correspondence, meeting notes, and artifacts that document the process were collected throughout the intervention to inform and contextualize dimensions of the intervention as guided by the RE-AIM framework.

### Data Analysis

Evidence-based practices were coded as nutrition or physical activity by the task forces proposing the changes. These practices were then coded to the sub-areas of changes by two researchers. Discrepancies in coding were identified and discussed by raters to determine the final coding. Interviews with LPHAs were transcribed and analyzed using structural, open, and axial coding (47, 48). Two researchers completed the analysis, with regular meetings to identify inconsistencies and discrepancies in coding and to discuss emergent themes (49). Project documents and records were analyzed by researchers to ensure the accurate and complete depiction of the intervention as it unfolded.

## Results

### Reach

**Figure 1:**
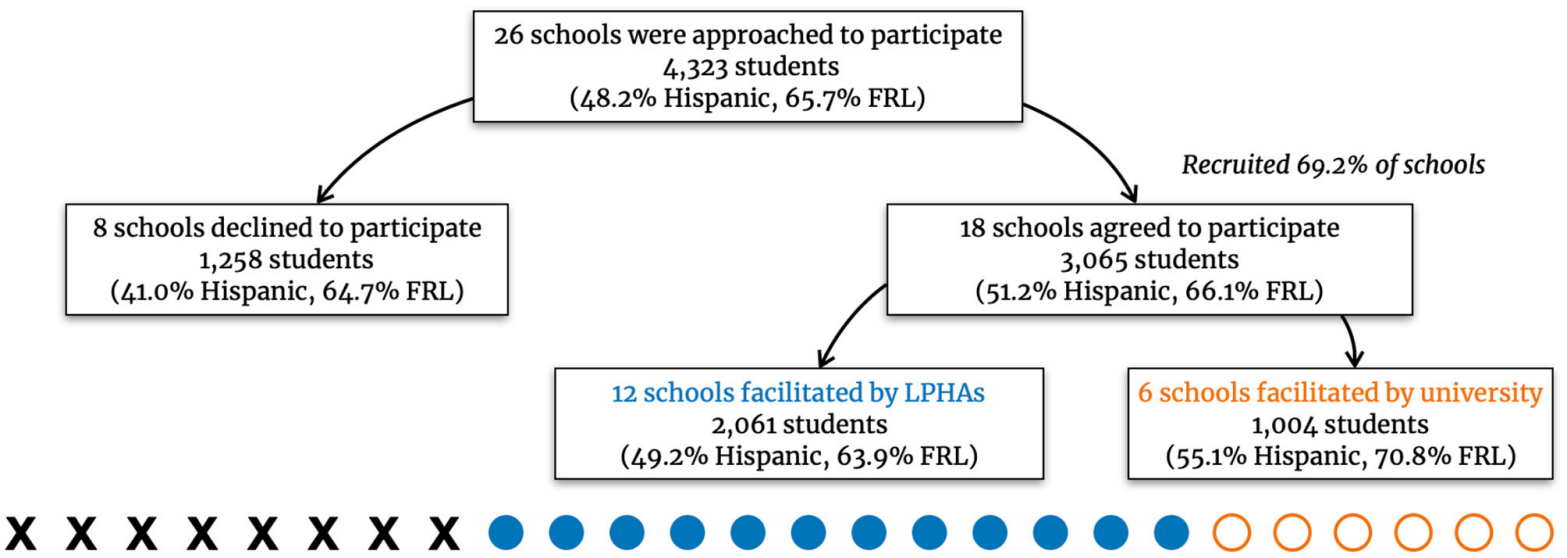
Demographics of participating and non-participating elementary schools.

**Figure 2:**
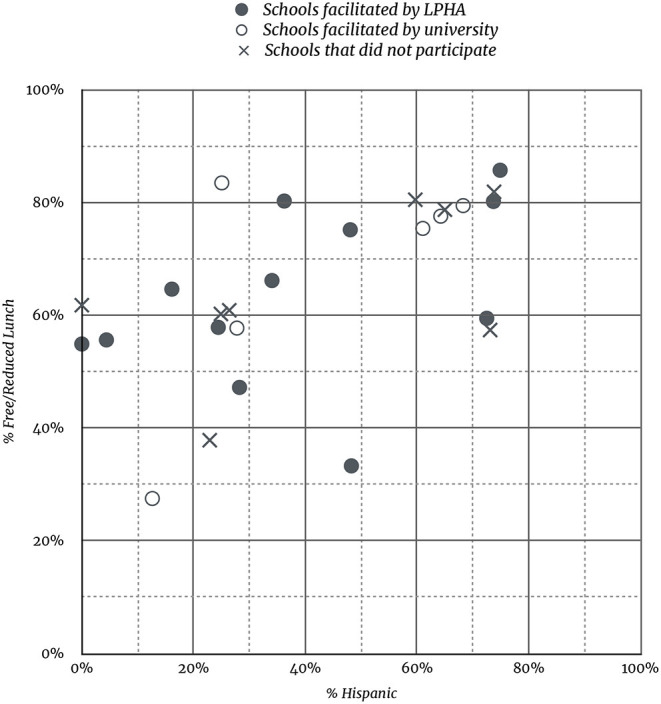
Scatterplot of student Free/Reduced Lunch rate and % Hispanic for participating and non-participating schools.

The target region for recruitment included 26 elementary schools. These schools served 4,323 students (48% Hispanic, 66% Free/reduced lunch). Among these schools, 18 (69%) agreed to participate and LPHA staff facilitated 12. A local individual was hired as university staff to facilitate the remaining six schools (see Figure 1). Schools that participated in the intervention as facilitated by LPHAs had a slightly higher Hispanic population (49%) and slightly lower free and reduced lunch rate (64%) than the target population (see Figure 2).

### Effect

The AIM process is designed to expedite the implementation of evidence-based practices that promote nutrition and physical activity for students at participating schools. The implementation status of identified practices was documented at the final meeting of the AIM process using the following options: fully implemented, partially implemented, planned for implementation, and not implemented.

**Figure 3:**
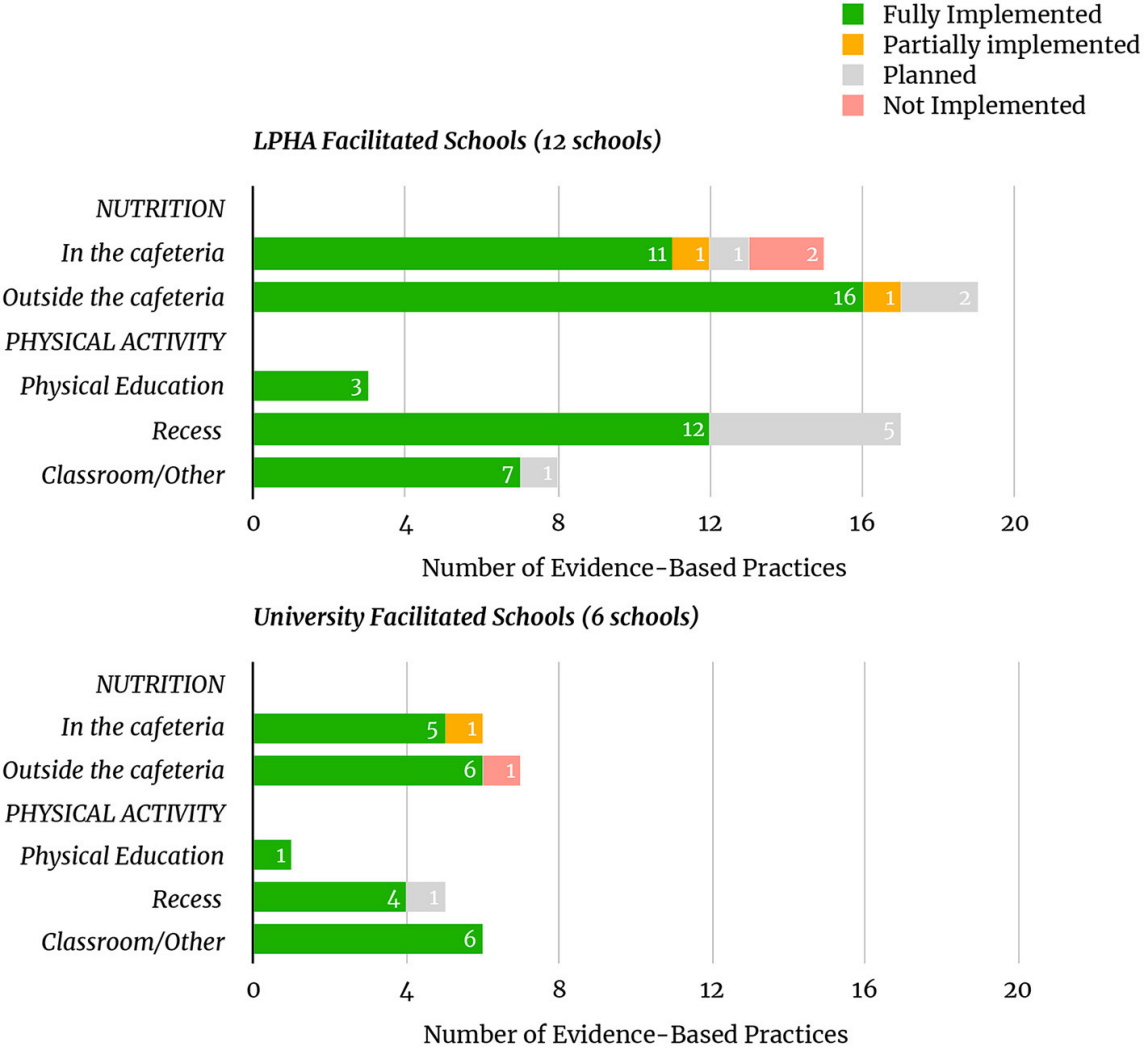
Number and implementation status of EBPs.

LPHA cohort 1 had an average of 5.20 changes implemented per school; LPHA cohort 2 had an average of 3.29 changes per school. The university-facilitated schools had an average of 3.67 changes fully implemented per school in both cohorts 1 and 2. In total, schools facilitated by LPHAs saw an average of 4.00 changes fully implemented per school, while schools facilitated by university staff had an average of 3.67 changes fully implemented per school. The results of the type of changes implemented are further delineated in Figure 3.

### Adoption

We attempted to recruit five LPHAs for partnership, and successfully recruited 100% of these LPHAs. In total, these five LPHAs serviced a population of 71,162 across seven counties. While all five LPHAs agreed to partner and implement AIM, two agencies were unable to identify personnel to facilitate all schools in their catchment area. Namely, one agency was able to facilitate just one of the six schools in their region, and another agency was able to facilitate one of the two schools in their region. Both LPHAs cited lack of available qualified personnel as the primary factor that limited their capacity to facilitate AIM in all schools in their regions. Among the 18 schools successfully recruited for participation in the process, the five LPHAs were able to facilitate 12 (67%) of those schools.

### Implementation

#### Number and Length of Meetings

In total, 94 meetings were scheduled with the 12 schools facilitated by LPHAs. Among these, 89 (95%) meetings took place. The six schools facilitated by university staff were scheduled for a total of 48 meetings, and 47 (98%) took place.

**Figure 4:**
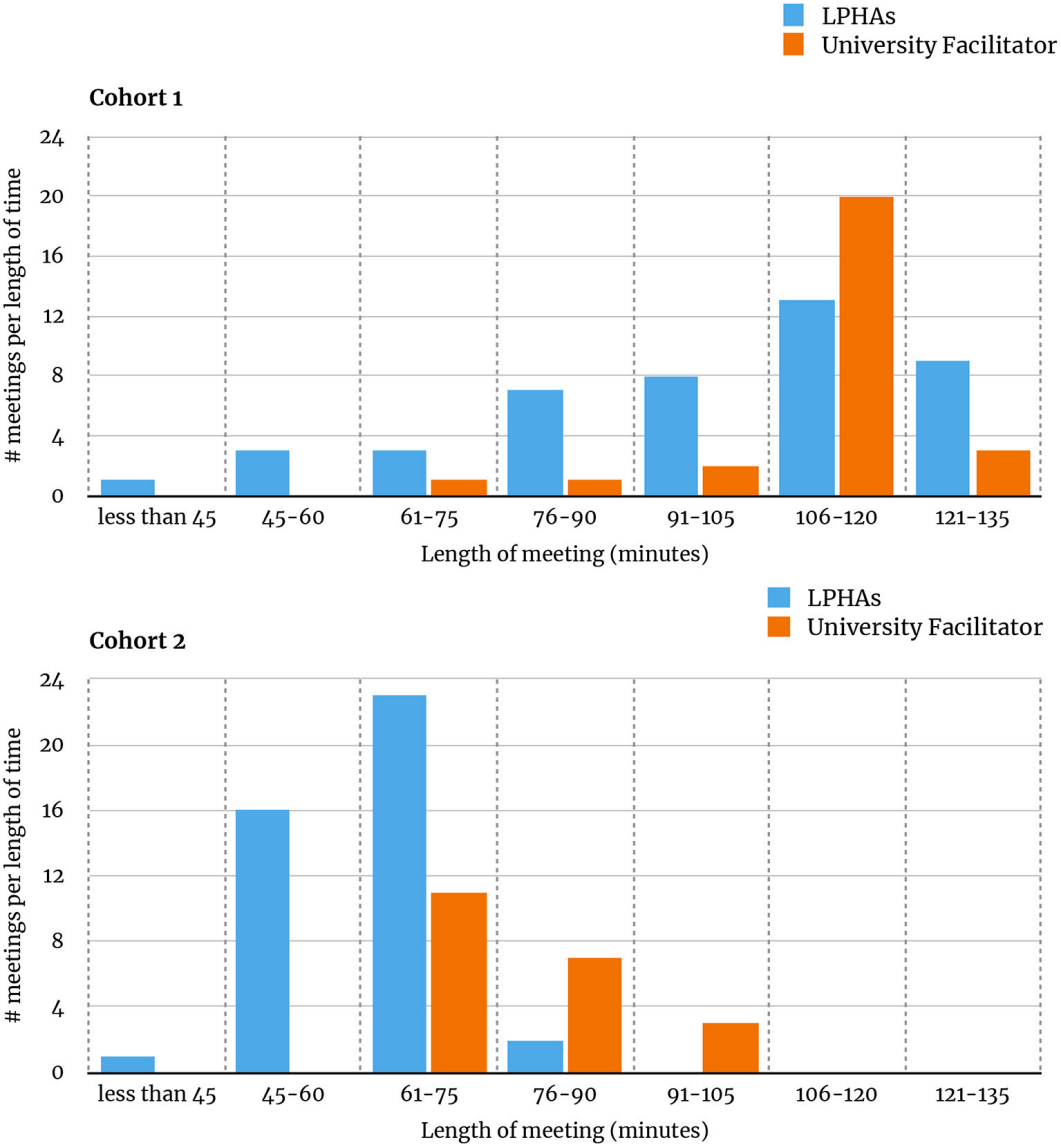
Length of meetings for cohorts 1 and 2.

Meeting lengths varied between cohorts 1 and 2 due to revisions made to the meeting guide based on feedback from cohort 1. There was no difference in mode for the meeting length between LPHA and university facilitators for either cohort (Cohort 1 mode = 1:46–2:00 h; Cohort 2 mode = 1:01–1:15 h). There was, however, a tendency for the university facilitator meetings to run longer than the LPHA facilitators across both cohorts. This was most pronounced during cohort 2 where the university facilitator meetings skewed longer (right) and the LPHA facilitator meetings skewed shorter (left; see Figure 4).

#### Time Spent Preparing; Feeling Prepared

Facilitators indicated how much time they spent preparing for each meeting. The university facilitator reported spending more than 60 min preparing for 77% of meetings while LPHA facilitators reported spending more than 60 min preparing for 50% of meetings (see Figure 5.1). Relatedly, the university facilitator strongly agreed with the statement “I felt very prepared to facilitate this meeting” for 94% of meetings while the LPHA facilitators strongly agreed with that statement for 39% of meetings (see Figure 5.2).

**Figure 5:**
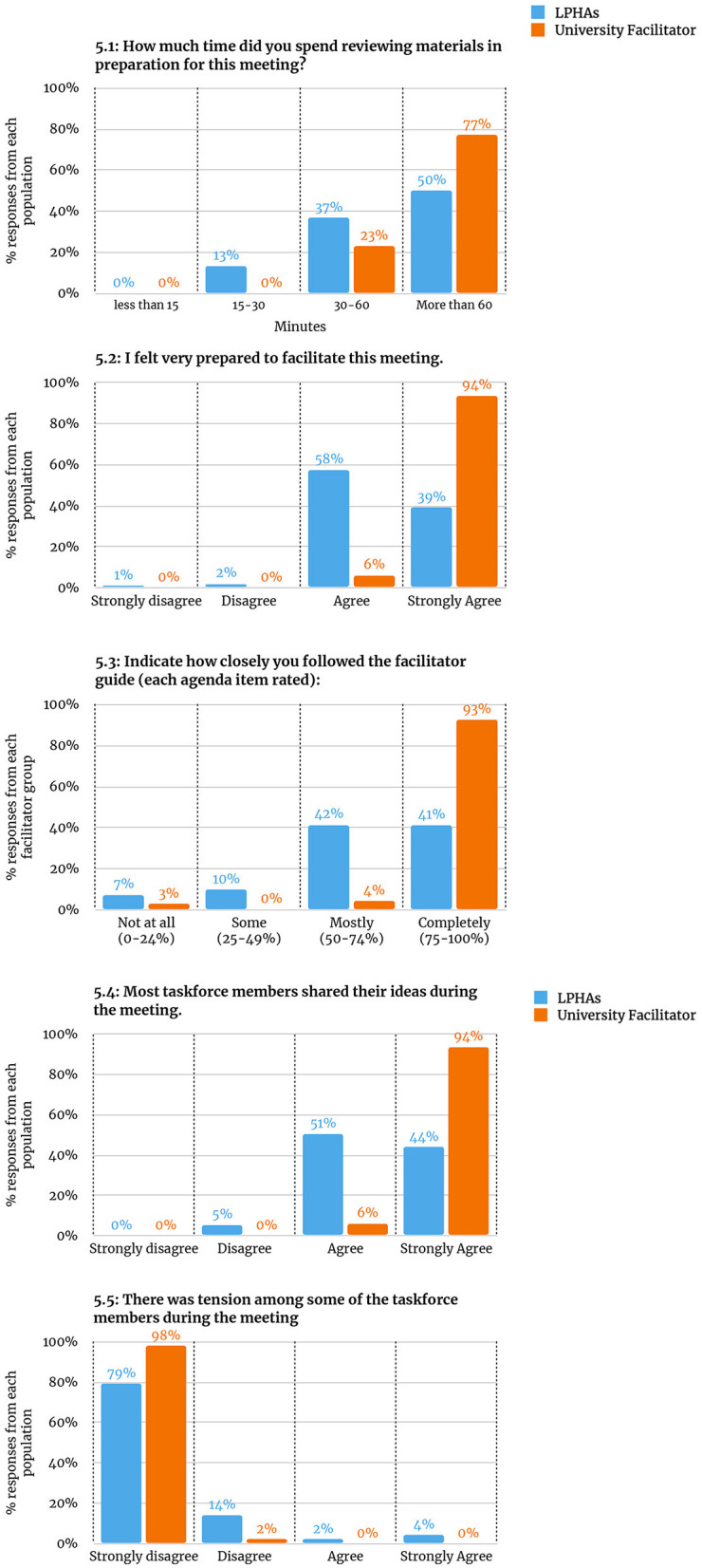
Facilitator ratings. **5.1:** How much time did you spend reviewing materials in preparation for this meeting? **5.2:** I felt very prepared to facilitate this meeting. **5.3:** Indicate how closely you followed the facilitator guide (each agenda item rated). **5.4:** Most taskforce members shared their ideas during the meeting. **5.5:** There was tension among some of the taskforce members during the meeting.

#### Fidelity to Facilitator Guide

Assess, Identify, Make it happen facilitators rated how closely they followed the facilitator guide for each agenda item of each meeting using the following scale: Not at all (0–24%, did not do this part of the meeting); Some (addressed 25%−49% of the items); Mostly (addressed 50%−74% of the items); Completely (addressed 75%−100% of the items). The university facilitator reported 93% of agenda items as “completely” while LPHA facilitators reported 41% of agenda items as “completely” (see Figure 5.3).

#### Idea Sharing and Tension During AIM Meetings

Facilitators also rated the extent to which they agreed or disagreed with two statements: “Most task force members shared their ideas during the meeting” and “There was tension among some of the task force members during the meeting.” The university facilitator strongly agreed that most task force members shared their ideas during the meeting 94% of the time, while the LPHA facilitators strongly agreed with this statement 44% of the time (see Figure 5.4). The university facilitator also strongly *disagreed* with the statement of tension among task force members 98% of the time, while LPHA facilitators strongly *disagreed* with this statement 79% of the time (see Figure 5.5).

#### Taskforce Satisfaction With the Process and Facilitators

At the end of the AIM process, task force members were invited to participate in a task force survey which included items focused on their satisfaction and interpretations of the AIM process (Figure 6.1) and facilitator (Figures 6.2, 6.3). These results show limited difference between satisfaction with the facilitator, although the LPHA-facilitated schools show slightly higher overall satisfaction with the process.

**Figure 6:**
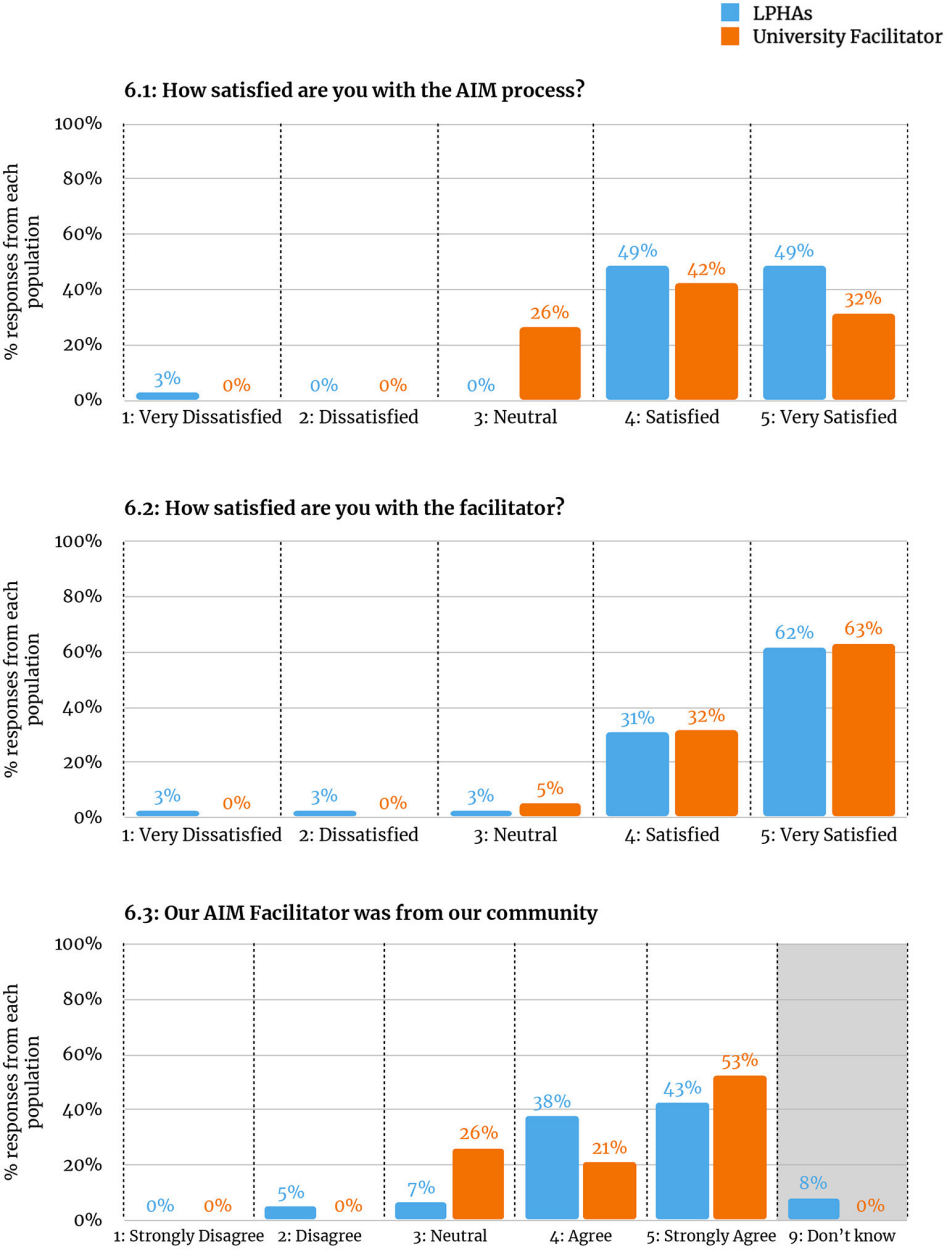
Taskforce member ratings. **6.1:** How satisfied are you with the AIM process? **6.2:** How satisfied are you with the facilitator? **6.3:** Our AIM Facilitator was from our community.

### Maintenance

The AIM process and partnerships with LPHAs resulted in several new connections and enduring practices amongst schools and LPHAs. At the close of the initiative, we offered an AIM Do-It-Yourself training and disseminated manuals for applying AIM without the support of a university facilitator. We did not systematically evaluate the uptake of such an approach at schools, however. Other outcomes from the initiative include school districts successfully transitioning AIM task forces into functional wellness teams, and LPHA staff continuing to meet with school district personnel to support them in their wellness efforts. Post-intervention interviews with LPHA staff also expressed optimism on the long-term outcomes for LPHA-school partnerships resulting from this initiative.

Relatedly, a subsequent iteration of AIM was offered 2 years after this initiative was completed in the same region. This version of AIM was altered in focus (from nutrition and physical activity to all components of the Whole School, Whole Community, Whole Child model) (50), scope (from school to district level), and implementation model (from nine, 60–75 min meetings, to three, 6 h meetings facilitated by University staff). Of the 16 school districts that agreed to participate in the initial version of AIM discussed in this study, nine (56%) also participated in this subsequent, extended version of AIM. Further, of the six districts that declined to participate in the initial version of AIM, 4 (67%) agreed to participate in the subsequent, extended version of AIM.

## Discussion

Implementing the AIM process in partnership with LPHAs allowed for a more scalable model of the AIM process to be implemented across a large, rural/frontier geographic region with outcomes comparable to previous iterations of AIM. This study raises a few points of ongoing consideration for those engaged in implementing interventions in partnership with local organizations as research intermediaries.

### Comparisons Between University and LPHA Facilitators of AIM

This study demonstrates that LPHAs succeeded in facilitating schools through the AIM process and that schools were successful in implementing EBPs. This positions AIM as a promising model for broader implementation to make schools in rural/frontier communities healthier places for students. There were, however, differences between LPHA and university facilitators in their facilitation of AIM in this initiative. The LPHA facilitators averaged lower marks than the university facilitator on (1) fidelity to the process, (2) the percentage of meetings that took place vs. those that were planned, and (3) the length and completion rate of meetings. Meetings facilitated by LPHAs also reported greater tension and lower incidence of all task force members sharing their opinions during the meetings when compared to meetings guided by the university facilitator. These differences are at odds with the outcome measures, which showed an average of slightly more evidence-based practices implemented with LPHAs (4.00 EBPs per school) than with the university facilitator (3.67 EBPs per school). These results support previous research that suggests intermediaries may be effective in facilitating the uptake of EBPs through community-engaged approaches (31, 32).

### Considerations of Fidelity

While fidelity is typically positioned as a key determinant to maintaining desirable outcomes of interventions, this study revealed that higher fidelity to the process as prescribed was not associated with an increased prevalence of desired outcomes (51). From a training and technical assistance perspective, our approach to fidelity was aligned with suggestions that an adaptive approach to fidelity is essential when scaling up programming (52). In this initiative, facilitators were encouraged to waver from the facilitator guide when they considered it in the best interest of the process and task force. In some instances, facilitators were supported in making more significant alterations to the process as long as critical activities of AIM were retained. Anecdotal evidence from this initiative supports the effectiveness of this adaptive approach to process fidelity. For example, there were instances in this implementation of AIM in which facilitators' high fidelity to the process was viewed as inflexibility to the local context and considered a detriment to quality by task force members. Conversely, approaching the AIM process with flexibility to the needs and contexts of LPHA and school partners was viewed as critical to ensuring the success of the initiative. These findings inspire a continued consideration of fidelity in the context of health-based interventions in partnership with community organizations in school settings (53).

### Importance of Effective Partnerships, Scalability Considerations, Training, and Technical Assistance

This study also emphasizes the benefits of adopting a flexible and supportive approach to partnering with community-based research intermediaries. In retrospect, we view approaches to (1) adapting to local capacity, (2) scalability, and (3) training and technical assistance, as worthy of emphasis.

#### Adapting to Local Capacity

Adapting the intervention plan based on the capacity of LPHAs was critical to ensuring success and promoting the greatest reach possible. For instance, although it was not the intended implementation model, we hired a community affiliate to operate as facilitator to account for the lack of available personnel in two LPHAs. Flexibility in implementation with this agency allowed us to still reach the target audience of schools in this region despite a lack of capacity at the LPHA.

#### Scalability

The effort to create a scalable model was executed with consideration of key dimensions of scalability [see (41)]. Revisions to the process that better positioned it for success in this scalable model include developing a new training and support model, revamping materials (meeting guide, website, supportive materials), amending the method of implementation (meeting evaluations, school surveys to generate automated reports), and, perhaps most importantly, reducing the amount of time required to complete the process. In the context of rural LPHAs and schools, it is important that initiatives that add to the existing workload honor the time constraints and responsibilities of existing partners and take efforts to promote the greatest efficiency possible. This approach was also more cost-efficient than previous versions of the process (29).

#### Training and Technical Assistance

Finally, many LPHA staff reported that the training and technical assistance they received throughout this intervention was both critical in aiding their successful facilitation and dissimilar to much of the training and support they had received in the past. This underscores the importance of attending to training and technical assistance when seeking to expand the reach of a model or intervention. In this case, a training and technical assistance approach that draws on various theories of education, training, and professional development was found to develop the necessary knowledge and skills in facilitators. This contributes to discourse concerning the importance of technical assistance in implementing new interventions and programs (54, 55).

## Conclusions

Implementing AIM with rural LPHAs as facilitators was an effective method of implementing evidence-based practices for physical activity and nutrition in rural elementary schools. The results outlined above support the continued exploration of partnerships with LPHAs as research intermediaries and the promise of further applications of AIM as a catalyst of expediting the research to practice delay.

Future studies may further engage in the question of fidelity in implementation science. Namely, the findings of this study support the importance of discourse that interrogates the notion of fidelity to interventions alongside responsiveness to the context and locality in which an intervention is implemented (51). Other research may address how partnerships with LPHAs can be leveraged and best structured to address areas of need in rural contexts (e.g., professional development needs, lack of funding, resources, or personnel) and promote positive outcomes to address a compendium of health behaviors and conditions.

## Data Availability Statement

The datasets generated and analyzed during this study are not publicly available due to considerations of confidentiality. De-identified selections of data may be made available from the corresponding author on reasonable request and in compliance with COMIRB.

## Ethics Statement

This study was reviewed and approved by the Colorado Multiple Institution Review Board (COMIRB). Participants provided their written informed consent to participate int his study.

## Author Contributions

BI, CL, and EB all had central roles in designing and implementing the intervention, collecting and analyzing data, and drafting the manuscript. All authors read and approved the final manuscript.

## Funding

This work was made possible with funding from the Colorado Health Foundation (PI: EB, Grant ID: 5733).

## Conflict of Interest

The authors declare that the research was conducted in the absence of any commercial or financial relationships that could be construed as a potential conflict of interest.

## Publisher's Note

All claims expressed in this article are solely those of the authors and do not necessarily represent those of their affiliated organizations, or those of the publisher, the editors and the reviewers. Any product that may be evaluated in this article, or claim that may be made by its manufacturer, is not guaranteed or endorsed by the publisher.
